# A longitudinal course pilot to improve surgical resident acquisition of quality improvement skills

**DOI:** 10.1371/journal.pone.0254922

**Published:** 2021-07-19

**Authors:** Vanita Ahuja, Jolanta Gorecka, Peter Yoo, Beth L. Emerson

**Affiliations:** 1 Department of Surgery, Yale School of Medicine, New Haven, Connecticut, United States of America; 2 Department of Pediatrics, Section of Pediatric Emergency Medicine, Yale University School of Medicine, New Haven, Connecticut, United States of America; Dalhousie University, CANADA

## Abstract

**Problem:**

Despite mounting evidence that incorporation of QI curricula into surgical trainee education improves morbidity and outcomes, surgery training programs lack standardized QI curricula and tools to measure QI knowledge. In the current study, we developed, implemented, and evaluated a quality improvement curriculum for surgical residents.

**Intervention:**

Surgical trainees participated in a longitudinal, year-long (2019–2020) curriculum based on the Institute for Healthcare Improvement’s online program. Online curriculum was supplemented with in person didactics and small group projects. Acquisition of skills was assessed pre- and post- course via self-report on a Likert scale as well as the Quality Improvement Knowledge Application Tool (QIKAT). Self-efficacy scores were assessed using the General Self-Efficacy Scale. 9 out of 18 total course participants completed the post course survey. This first course cohort was analyzed as a pilot for future work.

**Context:**

The project was developed and deployed among surgical residents during their research/lab year. Teams of surgical residents were partnered with a faculty project mentor, as well as non-physician teammates for project work.

**Impact:**

Participation in the QI course significantly increased skills related to studying the process (p = 0.0463), making changes in a system (p = 0.0167), identifying whether a change leads to an improvement (p = 0.0039), using small cycles of change (p = 0.0000), identifying best practices and comparing them to local practices (p = 0.0020), using PDSA model as a systematic framework for trial and learning (p = 0.0004), identifying how data is linked to specific processes (p = 0.0488), and building the next improvement cycle upon success or failure (p = 0.0316). There was also a significant improvement in aim (p = 0.037) and change (p = 0.029) responses to one QIKAT vignette.

**Lessons learned:**

We describe the effectiveness of a pilot longitudinal, multi component QI course based on the IHI online curriculum in improving surgical trainee knowledge and use of key QI skills.

## Introduction

The call to train physicians knowledgeable about and proficient in performance improvement is increasing. Specific to the developing surgeon, the Accreditation Council for Graduate Medical Education (ACGME) recognizes Systems-Based Practice as an essential competency milestone within the Improvement of Care practice domain [1]. Once in practice, the American Board of Surgery includes practice improvement as a dimension of Continuous Certification requirements [2]. Thus, developing these skills early on and refining them throughout one’s career is an expected part of surgical practice.

Achieving competency in performance/quality improvement (QI) practices has measurable benefit. Hospitals engaged in the American College of Surgeons National Surgical Quality Improvement Program (NSQIP) report reduced post-surgical adverse events, while a two-year NSQIP-based improvement program for residents was associated with reduced rates of pneumonia and surgical site infections [3, 4]. Many additional quality reports in the literature demonstrate improvements in morbidity and mortality with focused quality improvement efforts.

Further, quality improvement training provides positive benefits to individuals as well as the entire healthcare system. Studies suggest that provider quality improvement training is correlated with increased self-efficacy scores, which has a sustained benefit on physician well-being and is associated with increased effort and perseverance in the face of challenges [5, 6]. Thus, the positive effects of QI training on developing resilient, engaged physician warrant additional studies.

Surgical trainees have been engaged in improvement learning in a variety of ways, and numerous benefits of QI programs have been suggested in prior work. Participation in quality improvement curricula during surgical residency has been shown to improve self-reported knowledge and efficacy, while such curricula in surgical specialties have demonstrated that QI education is feasible, well-received, and improves QI related knowledge [7, 8].

In this study, we aimed to develop, implement, and evaluate a quality improvement curriculum for surgical residents to both teach QI and enmesh trainees in the improvement culture in our hospital system. A pilot first cohort was analyzed, to understand course performance and inform future curricular development. In addition to the knowledge of QI training provided by prior literature, we aimed to add a rigorous, validated evaluation of QI skill attainment and self-efficacy among participants.

## Methods

This study was performed in a cohort of surgical residents during dedicated research time (between PGY-2 and -3 in general surgery and plastic surgery) within an academic medical center. The curriculum was designed by a physician in local graduate medical education quality leadership, as well as a surgeon with quality experience. The faculty instructors had advanced quality improvement education through advanced course work, Institute for Healthcare Improvement, and Six Sigma. All participants were registered concurrently with the Institute for Healthcare Improvement’s online program, and achieved an Open School Basic Certificate during the 9 month course (www.ihi.org) [9].

The course began in September of 2019 and was completed in May of 2020. Concomitant with didactic work, the participants were paired in groups with a faculty mentor. Each group defined, initiated, and tracked a quality improvement project of interest. These included topics such as improving preoperative documentation of nutrition status for breast reconstruction surgery, reduction of loss to follow up following vascular procedures, improving documentation of ultrasound reads for pediatric abdominal pain, and improving clean closure practices in colorectal surgery cases. Participation in projects was used to highlight the quality improvement learning objectives throughout the QI curriculum.

Course participants met every other week for 2 hours for the first 3 months, to reinforce didactic teaching. Didactic content included a history of quality improvement, SMART aims, key driver diagrams, process mapping, run charts and variation, and patient safety. These didactics reinforced and built upon the Open School curriculum basics. The latter months involved meetings every 3–4 weeks for 1–2 hours each, where teams presented their work and received peer and mentor feedback. Additional didactics focused on local quality improvement leaders presenting their work with focus on interventions, reliability, and sustainability. These presentations included projects in NSQIP, SARS-CoV-2 Pandemic response, simulation, systems leadership, and healthcare economics.

Participants were surveyed at the beginning and completion of the course. Attainment of quality improvement skills was assessed by self-report on a Likert scale as well as use of the Quality Improvement Knowledge Application Tool (QIKAT), which is a standardized tool to assess application of core QI concepts [10]. Two standard case vignettes were presented with participants identifying aims, measures, and changes for each. Responses were scored 0–3 by two independent reviewers, separately and blinded to each other’s response, following the QIKAT rubric and were averaged between reviewers to obtain a final score for each response.

Self-efficacy scores were gathered before and after completion of the curriculum using the General Self-Efficacy Scale, which has been validated widely among a variety of populations [11]. This tool requires Likert responses 1–4 for each of 10 items, and can be used to gather a total sum score. Surveys were created and administered using Qualtrics XM (Qualtrics, Utah, USA). Pre- and post-curriculum scores were compared by Fischer’s Exact Test for self-reported skill acquisition and self-efficacy, and by t test for averaged QIKAT scores.

### Ethics

This study was deemed exempt by the Yale University Human Research Protection Program Institutional Review Board (Protocol ID 2000025869). As an exempted study, formal written or verbal consent was waived by IRB and not required from participants. At the beginning of the survey tool, participants were informed about study risks and investigator and IRB contact information.

## Results

A total of 18 residents participated in the QI course. Fourteen participants completed the pre-course survey, and 9 completed the post-course survey. Overall, residents self-reported an increase in acquisition of 8/11 key quality improvement skills (Table 1). Significant improvements were noted in studying the process (p = 0.0463), making changes in a system (p = 0.0167), identifying whether a change leads to an improvement (p = 0.0039), using small cycles of change (p = 0.0000), identifying best practices and comparing them to local practices (p = 0.0020), using PDSA model as a systematic framework for trial and learning (p = 0.0004), identifying how data is linked to specific processes (p = 0.0488), and building the next improvement cycle upon success or failure (p = 0.0316). A trend toward improvement was also noted in writing a clear problem statement (p = 0.0726).

**Table 1:** Difference in self-reported acquisition of skills pre and post QI course.

Question	Average (pre)	Average (post)	p value by Fischer’s Exact
Writing a clear problem statement (goal, aim)	2.43	3.22	*0.0726
Applying the best professional knowledge	2.5	2.89	0.3401
Using measurement to improve care	2.36	3.11	0.1927
Studying the process	2.21	.311	*0.0463
Making changes in a system	1.86	2.89	*0.0167
Identifying whether a change leads to an improvement	2.21	3.33	*0.0039
Using small cycles of change	1.71	3.33	*0.0000
Identifying best practices and comparing them to local practices	2.14	3.33	*0.0020
Using PDSA model as a systematic framework for trial and learning	1.36	3	*0.0004
Identifying how data is linked to specific processes	1.86	2.89	*0.0488
Building your next improvement cycle upon success or failure	1.79	3.11	*0.0316

(Not at All (1)/Slightly (2)/Moderately (3)/Extremely (4)).

*How comfortable are you in your current skills with the following aspects of quality assessment and improvement*?

Two validated QIKAT questions were administered pre and post course completion and scored separately. A trend toward improvement was observed across all responses, with significant improvement in aim (p = 0.037) and change (p = 0.029) responses for the second vignette (Table 2).

**Table 2:** Difference in QIKAT scores pre and post QI course.

*QIKAT–Question 1*	*Pre*	*Post*	*P value*
Aim	1.89	2.28	0.093
Measure	2.07	2.39	0.211
Change	2.07	2.39	1.96
*QIKAT–Question 2*	*Pre*	*Post*	
Aim	1.93	2.39	*0.037
Measure	2.39	2.61	0.323
Change	2.21	2.72	*0.029

Relative to self-efficacy, residents reported moderate levels of self-efficacy at the onset of the project, similar across most domains including problem solving, goal attainment, efficiency, resilience, and resourcefulness. While a trend toward increase was seen in nearly all categories (9/10 questions), the difference was not statistically significant (Table 3).

**Table 3:** Similar self efficacy scores pre and post QI course.

Question	Average (pre)	Average (post)	p value by Fischer’s Exact
I can always manage to solve difficult problems if I try hard enough.	2.71	3.11	0.4553
If someone opposes me, I can find the means and ways to get what I want.	2.71	3	0.2403
It is easy for me to stick to my aims and accomplish my goals.	2.93	3.11	0.7534
I am confident that I could deal efficiently with unexpected events.	3	3.11	0.7663
Thanks to my resourcefulness, I know how to handle unforeseen situations.	2.79	3	0.5522
I can solve most problems if I invest the necessary effort.	3	3.33	0.4171
I can remain calm when facing difficulties because I can rely on my coping abilities.	3	3.33	0.4171
When I am confronted with a problem, I can usually find several solutions.	2.71	2.89	0.5940
If I am in trouble, I can usually think of a solution.	3.14	3	0.5020
I can usually handle whatever comes my way.	3.14	3.44	0.6203

(Not at All True (1)/Hardly True (2)/Moderately True (3)/Exactly True (4)).

*Please select your agreement with each statement below*.

## Discussion

Quality improvement curricula for surgical trainees have been shown to improve surgical morbidity and national outcomes [4]. Despite these dramatic improvements, scholarship regarding general surgery training program incorporation of QI curricula lags behind that of other training programs and lacks standardization [12–14]. Although the optimal components of a QI curriculum have yet to be elucidated, and vary across disciplines, a combination of longitudinal didactics and experiential learning from small group projects has shown efficacy [15–17]. In the present study we describe a longitudinal year-long, multi component curriculum based on and inclusive of the Institute for Healthcare Improvement QI curriculum. Implementation of an online curriculum with structured in person didactics and small group QI projects resulted in self-reported improvement in 73% of key QI skills, including studying the QI process and making system wide changes via implementation of PDSA cycles. Thus, our intervention successfully improved resident knowledge of QI project implementation and evaluation.

Although QI curricula are becoming more widely implemented in the healthcare setting, few tools exist to reliably and consistently measure changes in QI knowledge and skills [18–20]. The QIKAT is a validated tool used to assess differences in QI knowledge after a training intervention and has previously been used to measure residency program curriculum success [18]. Here, we employed QIKAT to assess the efficacy of our novel QI curriculum and found a trend toward improvement across all responses, with significant improvement in responses to aim and change questions. These findings are consistent with self-reported improvement in QI skill knowledge, confirming QIKAT’s applicability in measuring change following our QI curriculum’s interventions.

Although previous studies conducted in psychiatry residency programs have found an improvement in QIKAT and self-efficacy ratings following a QI curriculum intervention, we did not detect significant differences in resident reported self-efficacy [14]. While residents reported moderate levels of self-efficacy across fields including problem solving, goal attainment, efficiency, resilience, and resourcefulness, these ratings were unchanged prior to and after our intervention. While this finding could suggest a lack of our intervention’s effect on self-efficacy, it could result from a lack of sensitivity in detecting self-efficacy as determined by a questionnaire.

Limitations of this study include small sample size, and single site of implementation. These issues may impact generalizability of our findings and may have impacted our ability to detect statistical significance. Participating residents were in research years, protected from most clinical activity, which is not characteristic of all resident experiences. Future directions include the expansion of this work to encompass a larger cohort of residents, and to evaluate for transferability between institutions. Additionally, future work could evaluate the sustainability of the knowledge attainment and perhaps further involvement in quality work.

Unique features of this work are several. We incorporated not only didactic teaching, but immersion in experiential project-focused mentored learning throughout the course. This was coupled with an emphasis on working in multi-disciplinary teams, and most projects included non-physician team members. Although the project represents a small pilot, the rigorous approach to curricular assessment in QI is distinctive. We leveraged not only self-report, but also a validated tool to assess attainment of curricular objectives. Similarly, we demonstrate the feasibility of the assessment of self-efficacy in this setting. The observed non-statistically significant trend towards improvement suggest that this is an important future direction for exploration in QI education.

## Conclusion

In conclusion, our study is amongst the first to describe the effectiveness of a longitudinal course composed of a formulated online curriculum, didactics, and small group practical QI initiative projects in improving surgical trainee knowledge and use of key QI skills as measured both by self-report and QIKAT. Though our study was limited by a small sample size from a single institution general surgery program, without evaluation of the longevity of acquired skills, it demonstrated marked improvement in participant QI knowledge.

## Supporting information

S1 Data Surgery resident QI program—Raw data. (XLSX)
